# Skin and Genito-Urinary Diseases

**Published:** 1893-05

**Authors:** 


### SKIN AND GENITO-URINARY DISEASES.

EDITED BY DR. M. B. HUTCHINS.

### TREATMENT OF SOFT CHANCRE. (CHANCROID.)

Dr. Brocq (Paris), in correspondence to March *Journal Cutaneous and Genito-Urinary Diseases,* gives the following as Balzer's treatment of chancroid:

1. The most scrupulous care as to cleanliness, as complete asepsis as possible about the virulent focus, and in this respect the local hot baths recommended by Dr. Aubert cannot be too highly spoken of. These have a temperature of over forty degrees (C.). 2. After the local bath, caustic application with solution of chloride of zinc, nitrate of silver, phenic acid, etc., or as the author does in his service, apply a paste containing one part of chloride of zinc, nine parts of oxide of zinc and a quantity of water sufficient to make into a paste. These caustics are to be applied as frequently as is necessary to destroy the virulence of the chancre and transform it into a simple wound. 3. When this effect has been obtained, and in the intervals of the caustic applications, permanent dressings with feeble antiseptics in powder or in solutions, and for this purpose are employed iodoform, aristol, nitrate of silver, etc. Until complete cicatrization the patient must keep as quiet as possible and avoid all irritating contacts which might aggravate the chancre or provoke the appearance of bubo. For my own part, in simple cases the treatment which I have always seen succeed the best is: 1. Washing morning and night with boric acid water as hot as possible. 2. Applications once a day with a nitrate of silver solution, one in twenty. 3. Dressing rigorously carried out with powdered iodoform. 4. A protecting covering well made.

### DERMATALGIA.

Dr. Geo. T. Elliott mentioned at the 220th meeting of the New York Dermatological Society (March *Journal Cutaneous and Genito-*

*Urinary Diseases*) a case of this disease persisting four months, having followed an attack of rheumatism in a gouty patient. From the inguinal folds to both knees was so senistive that the slightest touch or rubbing produced intense pain. General health was good. He mentioned a second case, probably the result of neurasthenia following typhoid fever. The editor has had two attacks of this disorder, in the beginning of cool weather, when in something of a rheumatic condition. There was a constant sensation as if the skin had been scalded or blistered, the contact of the clothing or the slighest touch aggravating the sensation—the hairs seemed as if each was set in a boil. Firm pressure produced no sensation, only the slight touch being responded to by the terminal sensory nerves. The condition is probably neuralgic. Constitutional treatment, and galvanism to the affected area seem indicated.

---

### THE ORIGIN OF TERTIARY SYPHILIS.

Dr. A. Haslund, of Copenhagen, has a clinical article upon this subject in Band XVI., Nu. 3, Monatshefte für Praktische Dermatologie. A brief mention of the article can only be given here. He believes that the majority of the so-called tertiary lesions have their seat in the situation of an earlier lesion, may even occur upon the site of the chancre. He says that the occurrence of gummata over the most superficial bones is due to their being more exposed to injury. He thinks people who exercise the body most are more subject to body (as skin) late lesions, while syphilis of the nervous system is more common in the higher walks of life, where the nervous system and brain are more exercised. Vaccination frequently excites an attack of late syphilis, often starting at the inoculated site.

---

### CANCER OF THE TESTICLE IN A CHILD.

In Band XVI., No. 5, of the above quoted journal, is a reference to a case reported by Sabrazes and Fromaget (Bordeaux). A child of two and a half developed, eight months before, a tumor the size of a mandarin, in the left side of the scrotum, hard, of slight sensibility. Castration was followed by recovery. The tumor was an epithelial carcinoma with a small amount of connective tissue development. The authors knew of twenty-six similar cases in literature; two of enchondroma, the remainder carcinoma or sarcoma. Prognosis unfavorable. Recurrence and death in six to twelve months.

### CORN STARCH.

Powdered corn starch, as used in cooking and other domestic ways, is one of the very best cooling applications for an irritated skin, as it absorbs moisture very rapidly, increasing cutaneous transpiration and cooling by evaporation. As Unna says *man friert sich* when so covered.

### THE BIRTH OF A SYPHILITIC CHILD FROM A HEALTHY MOTHER.

Immunity, rather than latent syphilis by *choc en retour*, is thought by Neumann to be the condition of mothers who bear syphilitic children and remain themselves free from signs of the disease.—*Journal of Cutaneous and Genito-Urinary Diseases, March,* 1893.

Pseudo-chancre redux or chancre-like syphiloma of Fournier is a late manifestation reproducing absolutely the appearances of an initial lesion, and as it is sometimes accompanied by a generalized eruption, has been mistaken for an evidence of reinfection. According to Morel-Lavallée the lesion is chronologically a secondo-tertiary one and is of the nature of a tuberculo-gummous syphiloma. —*Journal Cutaneous and Genito-Urinary Diseases.*

